# Safety Culture Measurement Among Chinese Undergraduates at a Private University: Development and Validation

**DOI:** 10.3389/fpubh.2022.825106

**Published:** 2022-03-28

**Authors:** Shan Gao, Chen Chang, Fang Ren, Fei Yu

**Affiliations:** ^1^School of Civil Engineering, Xijing University, Xi'an, China; ^2^School of Civil Engineering, Harbin Institute of Technology, Harbin, China

**Keywords:** safety beliefs, safety behaviors, private universities, Chinese universities, questionnaire

## Abstract

Relatively low level of safety culture among undergraduates and the imperfection of safety management system for students in university result in numerous safety problems. Researches on the safety culture of undergraduates in public universities are much more than those in private universities. Aiming to find out the potential and specific factors that affect the safety culture in the former are different from those in the latter, an anonymous questionnaire survey was conducted among 4,531 students in a private university in Shaanxi province, China. Gender, education background, grade, hometown, one-child policy, major, community, and driver's license on their safety beliefs and behaviors are treated as potential factors in the survey. According to the average score of each item, the investigated private university students are lack of safety knowledge, but perform well in traffic safety behavior. The results show that female students show better safety beliefs and safety behaviors than male students whilst the safety beliefs and safety behaviors of the student majoring in medicine is better than those of students in other majors. The students who live in more developed cities, who are from one-child family and who have driving license, show better safety beliefs and safety behaviors than others. The effective community system of mixed majors is conducive to the formation of good safety beliefs and safety behaviors of college students. The results highlighted that universities should formulate the corresponding intervention strategies to prevent safety problems of the college students according to the specific proportion of gender, major distribution and other actual situation.

## Introduction

With an increasing number of students in universities and many complex factors leading to disasters, the cultivation of safety beliefs and safety behaviors has been getting increased attention in universities. However, at the present stage, relatively low level of safety culture among undergraduates and the imperfection of safety management system for students in university result in numerous safety problems. In recent years, many safety accidents in campus have been reported at home and abroad, such as dormitory fire (killing 41 undergraduates and injuring nearly 200 undergraduates in the dormitory building of Peoples' Friendship University of Russia in 2003^1^), terrorism (killing 33 undergraduates and teachers in Virginia Tech which is the most serious campus shooting in the United States so far in 2007^2^), lab explosion (killing one undergraduate and injuring four in a laboratory explosion at China University of Mining and Technology in 2015^3^). These catastrophic accidents remind universities the importance of safety belief and behavior among the undergraduates.

Safety belief is the most basic idea to personal judgement in safety. Dong et al. (1) had made a classification summary of the accidents that occurred in universities from 2010 to 2015. The results show that the main factors that lead to safety accidents are the lack of safety belief. Jing and Wu (2) pointed out that the university students have a strong belief in safety, but their crisis awareness is relatively weak. Feng et al. (3) put forward the component relational network model using social network analysis (SNA) to improve the security management mechanism of universities. Yang and Li (4) identified the causes of safety accidents in universities through Systems-Theoretic Accident Model and Process (STAMP) accident model and process method.

Safety behavior depends on safety belief, but it is hard to turn safety belief into safety behavior easily. Walters et al. (5) pointed out that although students have a belief of safety, there are deficiencies in identification and emergency response when hazards occur, which need more guidance from universities to improve safety behavior of students. Wu et al. (6) and Qin (7) proposed that safety accident experience and safety training have practical significance on safety behaviors, and suggested that universities should carry out regular safety training to improve students' response ability. Li et al. (8) revealed various factors affecting safe behavior of college students. Thamrin et al. (9) concluded that many students working part-time off campus are prone to traffic safety accident. It should be mentioned that different from foreign universities studied by Thamrin et al. (9), most of the college students in Chinese universities live in campus. Reesi et al. (10) studied the risk driving behavior of students in Oman University, and concluded that it is necessary to integrate road safety into the field education.

At present, most of the researches on the safety culture of undergraduates are based on public universities. In the past decades, private universities in China have been developing rapidly, but still in their beginning stage. On the contrary, the number of the students in private universities of China is actually large. Relatively lower entrance threshold to private universities in China may lead to different safety culture level among the students, compared to those in the public universities. According to the statistical results of Wang and Wu (11), researches on the safety culture of undergraduates in public universities are much more than those in private universities. Moreover, the sample size of field survey on the safety culture of undergraduates is usually small.

Aiming to find out more potential and specific factors that affect the safety culture in Chinese private university and the difference between public university and private university with larger sample size, this study focuses on the safety culture (beliefs and behaviors) among Chinese undergraduates in a private university of Shaanxi province, which is the largest one in the northeast of China. The data is collected through anonymous online questionnaire survey. The correlation between the basic information and safety culture is by ANOVA and SPSS26 was used for data analysis. The reliability and validity evaluation are conducted through Cronbach's α and factor analysis, respectively. After analyzing the significant factors influencing the safety culture of private college students, some suggestions are made to improve the safety culture of private college students.

## Methodology

### Instrument

Before the formal investigation, the professors whose specialties involved safety education at universities and who acquainted with the research in safety culture area were invited to participate in the development of the questionnaires items. After the trial and first-run exploratory factor analysis, some the ambiguous and offensive items related to personal finances and religious belief were deleted. The items were guaranteed to not load at any factors or loading not exceed 0.4 in the factor analysis. Total 39 items related to the safety beliefs and safety behaviors of undergraduates were obtained as listed in Table 1, which include 8 items related to safety knowledge, 8 items related to interaction safety behavior, 8 items related to traffic safety behavior and 15 items related to campus safety behavior. A five-point Likert scale (1-Never, 2-Rarely, 3- Sometimes, 4-Most, 5-Always) was used to quantify the measurement of each item.

**Table 1:** Safety culture items and basic information of undergraduates in a private university.

**Category**	**Project**	**Item**
Basic information of the respondents	1.Gender	Q1
	2.Grade	Q2
	3.Hometown	Q4
	4.Education background	Q5
	5.Major	Q6
	6.Community	Q7
	7.Only child	Q8
	8.Driver's license	Q9
Safety knowledge	9.Do you often imagine yourself encountering safety problems and think about some countermeasures?	QA1
(self-thinking and learning)	10.Do you pay special attention to the news of terrorist attacks and learn some self-defense measures?	QA2
	11.Do you pay special attention to the fire news and learn some fire escape knowledge?	QA3
	12.Do you pay special attention to the earthquake disaster news and learn some escape methods?	QA4
	13.Do you pay special attention to food safety issues and learn some food safety knowledge?	QA5
	14.Do you actively participate in safety training courses or activities organized by the university?	QA6
	15.Do you deliberately avoid construction sites?	QA7
	16.When you come to a strange place, will you deliberately observe the location of fire equipment and safety exit?	QA8
Interaction safety behavior	17.Do you alert others when they are engaging in dangerous behavior or using dangerous substances?	QD1
	18.Do you double-check your belongings in crowded places?	QD2
	19.Do you take the initiative to smooth over the conflict with your roommate or classmates?	QD3
	20.When you suffer from mental stress or distress, do you talk to your teachers or classmates?	QD4
	21.Are you particularly concerned about people who behave strangely around you in crowded places?	QD5
	22.When you travel to strange places, do you look for some companions?	QD6
	23.When you go out alone, do you deliberately tell your roommates or friends where you are going?	QD7
	24.Before saying yes to a stranger's request to borrow your change or use your cell phone, do you worry about the stranger's true intentions?	QD8
Traffic safety behavior	25.When walking down the street, do you always observe and pay attention to traffic or other safety conditions around you?	QJ1
	26.Do you check traffic to the left and right when crossing the street?	QJ2
	27.Do you wait for traffic lights when no vehicle is passing?	QJ3
	28.When you're alone walking or riding a bike, do you keep focused, instead of listening to audio or thinking?	QJ4
	29.Do you wear helmet and other protective gear when riding a bike/e-bike/motorcycle?	QJ5
	30.Do you deliberately avoid going the wrong side of road when riding a bike/e-bike/motorcycle?	QJ6
	31.Do you prefer regular taxis and public transportation to carpooling and Uber?	QJ7
	32.Do you always use seat belts while driving or riding in a vehicle?	QJ8
Campus safety behavior	33.Do you always advise other students to avoid illegal campus loans?	QX1
	34.Do you pay special attention to the news of campus safety incidents?	QX2
	35.Do you pay as much attention to the safety on campus as you are off campus?	QX3
	36.Do you avoid walking alone at night no matter on and off campus?	QX4
	37.Do you strictly follow laboratory rules, even if it may cause inconvenience?	QX5
	38.Do you deliberately avoid using high-power electrical equipment in your dormitory?	QX6
	39.Do you keep your hands dry when you plug electrical equipment in or out?	QX7
	40.Do you deliberately lock your closet in your dorm room?	QX8
	41.Do you lock the door when you leave the dorm room for a short time?	QX9
	42.Do you check the switch of electrical equipment when you leave the room?	QX10
	43.Do you avoid running into or out of an elevator that is closing?	QX11
	44.Do you walk on the right side of the stairs?	QX12
	45.Do you pay special attention to fire equipment and evacuation exits in your daily study or living places?	QX13
	46.Do you avoid stimulating or dangerous sports and activities?	QX14
	47.Do you warm up before exercise (swimming, ball games, etc.)?	QX15

In addition, 8 personal basic information items of the undergraduates, including gender, educational background, grade, hometown, one-child policy, major, community, and driver's license, were collected through questionnaire to investigate how much the correlation between the basic information and safety culture is by ANOVA. SPSS26 was used for data analysis.

It should be mentioned that the community system is a type of management mode in universities. In the private university of this research, six communities were involved, including Major-Mixed community 1 (MM-C1), Major-Mixed community 2 (MM-C2), community preparing for the postgraduate entrance test (PPE-C), Art community (A-C), Liberal arts specialty community (LAS-C) and Science specialty community (SS-C). The last two are for junior college education.

### Subjects

The survey was carried out in the largest private university in the northwest of China. Formal survey was conducted in the form of online questionnaire distributed randomly through mobile APP. The survey time was from June 1, 2020 to June 24, 2020. Four thousand five hundred and thirty-one online questionnaires were collected. According to the principle of 3σ and normal distribution, a total of 53 abnormal questionnaires with <70 s or more than 490 s answer time and most of the missing data were excluded. In that case, 4,478 valid questionnaires with an effective response rate of 98.83% were obtained finally. Table 2 shows the summary of questionnaire processing.

**Table 2:** Summary of questionnaire processing.

	**Number**	**%**
Effective quantity	4,478	98.83
Exclude	53	1.17
Total	4,531	100.00

### Reliability and Validity Evaluation

#### Reliability

Cronbach's α ranging in 0–1 (12) is used to measure the internal consistency reliability coefficient of the questionnaire items of the five level Likert scale. Cronbach's α could be described as Equation (1):


(1) $$\alpha \;=\frac{k}{k-1}\left(1-\frac{\sum {s_{i}}^{2}}{{s_{i}}^{2}}\right)$$

where *k* is the total number of items in the scale. ${s_{i}}^{2}$ is the intra-class variance of the score of the question *i*. ${s_{i}}^{2}$ is the variance of the total score of all items.

Table 3 shows the evaluation principles of internal consistency coefficient based on the research of Nunally and Bernstein (13) and DeVellis (14).

**Table 3:** Evaluation principles for Cronbach's α.

**Cronbach's α**	**Used for a global scale analysis**
<0.50	Awful, abandoned
0.50 0.59	Bad, should be modified
0.60 0.69	Reluctantly acceptable, should be modified
0.70 0.79	Acceptable
0.80 0.89	Good
>0.90	Excellent

Cronbach's α of the questionnaire in this study is calculated through SPSS 26. It should be noted that 8 basic information items are not involved in the measurement of the scale and it is not necessary to conduct the reliability test and validity test of those items (15). As Table 4 lists, the Cronbach's α for 39 items in the questionnaire is 0.961, which is “Excellent” grades listed in Table 3 which indicates that the questionnaire in this study can be used for follow-up analysis.

**Table 4:** Reliability of the items related to safety culture.

**Cronbach's α**	**Number of items**	**Reliability evaluation**
0.961	39	Excellent

#### Validity

Factor analysis is the most commonly used statistical method for validity evaluation. Before conducting factor analysis, Kaiser-Meyer-Olkin's Measure of sampling adequacy (KMO) and Bartlett's Test of Sphericity should be conducted in SPSS. The evaluation principles of KMO are shown in Table 5 (16).

**Table 5:** Evaluation principles of KMO.

**KMO value**	**Fit degree description of factor analysis**
<0.50	Unacceptable
0.50 0.59	Miserable
0.60 0.69	Mediocre
0.70 0.79	Middling
0.80 0.90	Meritorious
>0.90	Marvelous

Table 6 presents that the KMO value for questionnaire is 0.967, which is “Marvelous” grade listed in Table 5. The chi-square test value in Bartlett's test of sphericity is 111102.743 which is relatively large. The corresponding significance is 0.00 < 0.05 at which level, the original hypothesis can be rejected. It shows that the possibility of sharing common factors among items is low, which is suitable for factor analysis.

**Table 6:** KMO and Bartlett's sphericity test results of the questionnaire.

**Test**		**Value**
Kaiser-Meyer-Olkin Measure of Sampling Adequacy		0.967
Bartlett's Test	Approximate Chi-Square distribution	111102.743
of Sphericity	Degrees of freedom	1176
	Sig.	0.00

In the results of factor analysis, the main indicators used to evaluate the validity are commonality and cumulative variance contribution rate. If the commonality is >0.4, it is acceptable. The cumulative variance contribution rate is more than 50%, which indicates that the effect of factor analysis is good.

According to Table A1 in Appendix A, the commonality of items in the corresponding factors is >0.4, which shows that the extracted factors can well reflect most of the information of the original variables. According to Table A2 in Appendix A, the cumulative variance contribution rate of the questionnaire is 59.329% > 50%. It shows that the five parts of the questionnaire can extract most of the item information, which indicates that the validity of the research data is good.

## Results

**Figure 1:**
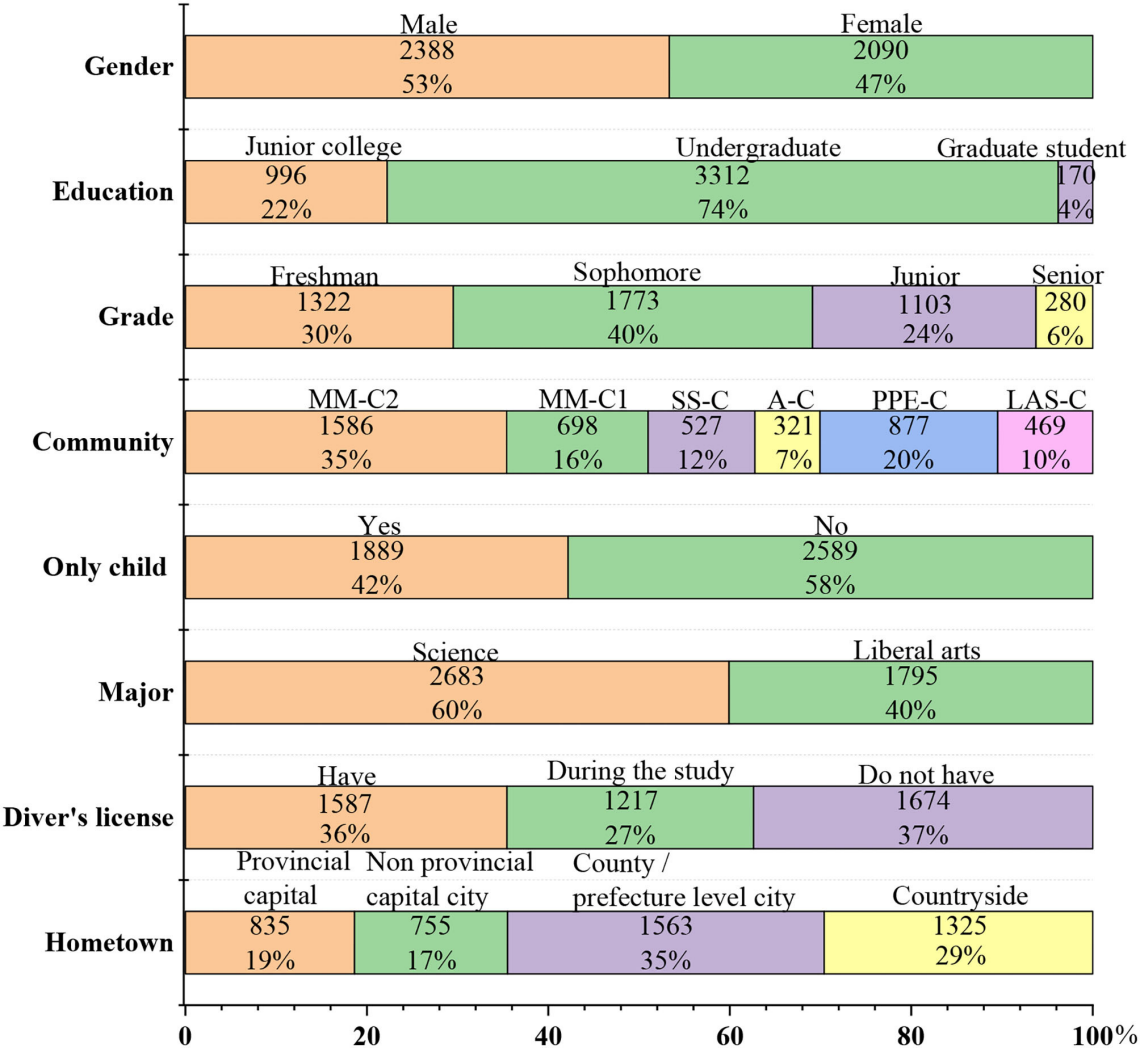
Basic information of respondents.

Figure 1 shows the basic information of respondents. In particular, regarding community, 16, 35, 20, and 7% of the respondents are MM-C1M (Major-Mixed community 1), M-C2 (Major-Mixed community 2), PPE-C (Community preparing for the postgraduate entrance test), and A-C (Art community), respectively, whilst 10 and 12% of the respondents are LAS-C (Liberal arts specialty community) and SS-C (Science specialty community) students, respectively. Basic information generally maintains a proportionate balance. As a result, the questionnaire which is effective and credible can be used for ANOVA.

**Figure 2:**
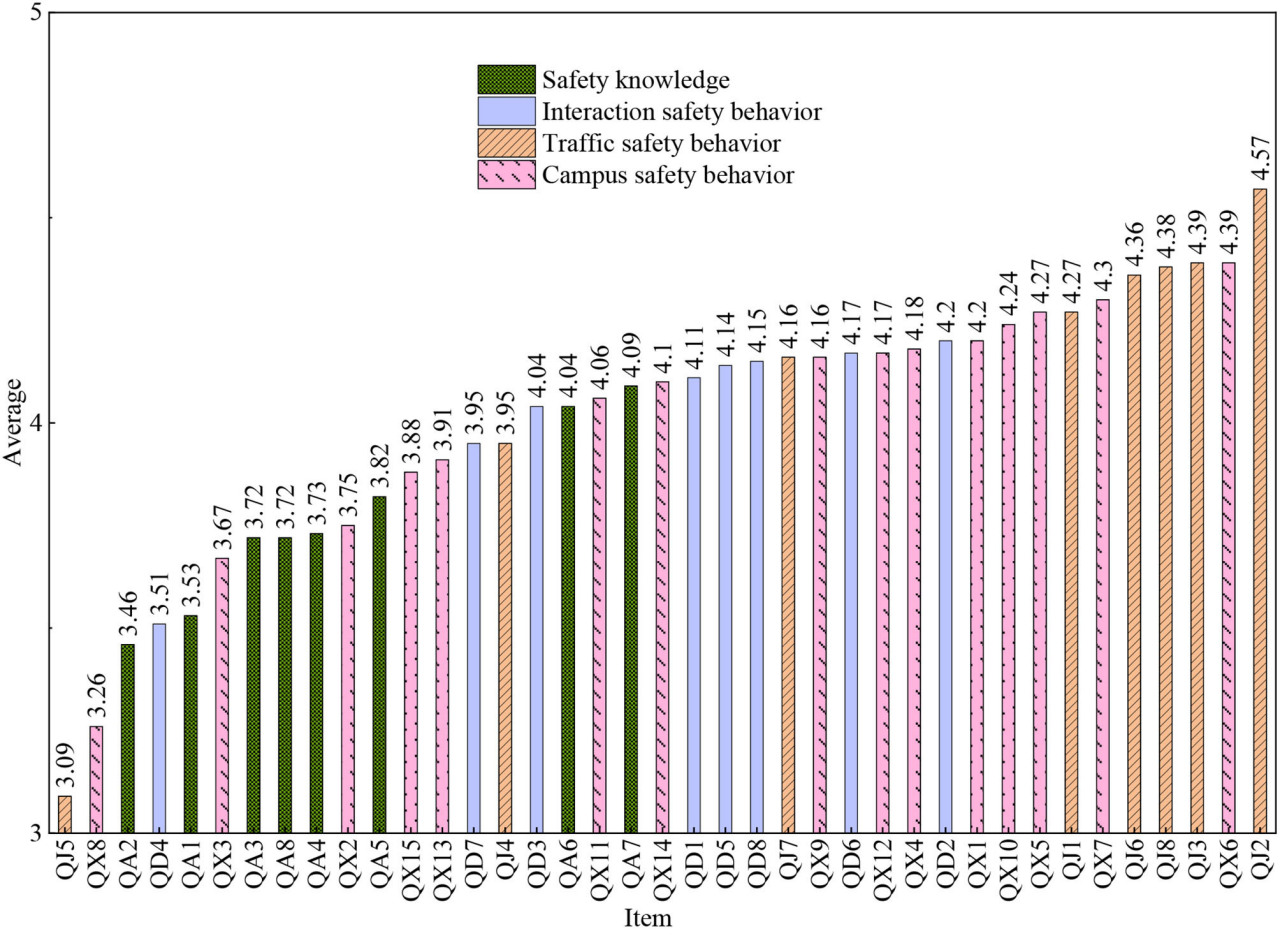
Ranking of mean of each item.

Figure 2 shows that the score of the items related to safety knowledge in this study is low, indicating that the safety knowledge of the investigated students is not enough. QJ5 (Do you wear helmet and other protective gear when riding a bike/e-bike/motorcycle?) gets lowest scores in this research. This may result from that young people tend to find it inconvenient or uncomfortable to wear helmets. In the research of Blair et al. (17), up to 71% of the respondents did not wear helmets, however wearing helmets can reduce the risk of serious head injury by 85%. It is not only necessary for the government to improve the policies to punish the behaviors violating the road safety rules, but also to strengthen the safety culture norms of students, so as to eliminate the practice of engaging in unsafe driving. QX8 (Do you deliberately lock your closet in your dorm room?) gets the second lowest scores in this research. This may result from the fact that the overall safety environment of the investigated private university which runs closed campus management, is quite good. The respondents tend to trust their roommates. In addition, the respondents may also feel that locking their own closet will show distrustful gesture to the roommates. The reason for the third lowest score of question QA2 (Do you pay special attention to the news of terrorist attacks and learn some self-defense measures?) may be that the gun control and anti-terrorism policies of Chinese government are quite restrict, resulting in excellent social stability and environmental security in China. The fourth lowest score of QD4 (When you suffer from mental stress or distress, do you tell your teachers or classmates?) may be due to the fact that college students are commonly sensitive and vulnerable. They are unwilling to share their troubles with others, worried about being discriminated by teachers and classmates, instead of being understood, namely “mental health stigma problem”. It may also be that Chinese people are introverted and prefer to talk to their relatives. It is suggested that universities should strengthen propaganda to reduce mental health stigma problem among college students, letting them accept psychological counseling and establish correct cognition of psychological help (18, 19).

On the contrary, the respondents did better in the following items. The scores of QJ2 (Do you check traffic to the left and right when crossing the street?) and QJ3 (Do you wait for traffic lights when no vehicle is passing?) are very high. The popularization of traffic safety education in primary and secondary schools has played a good role in the safety behavior of college students. Meanwhile, it may be that when college students cross the road, they would be affected by the people around them who obey the traffic rules (20). The score of QX6 (Do you deliberately avoid using high-power electrical equipment in your dormitory?) from Chinese private university students in this study is high, whilst in the investigation of Hasan and Younos (21), the awareness of foreign students on electricity safety is very poor. This high score of QX6 reflects the good effect of the apartment management work on banning the use of illegal electrical equipment in Chinese universities. Certainly it is also possible that the students who answered the questions were afraid of being found to have violated this rule, and intentionally choose “always”. The high score of QJ8 (Do you always use seat belts while driving or riding in a vehicle?) may be due to the relevant laws on seat belts issued by China's transportation department, which force the front-row passengers to use seat belts. If not, they will be fined and even affect the validity of their driver's license. It can be seen that the mandatory traffic laws could directly affect the traffic safety beliefs and behaviors of people.

## Discussions

SPSS26 is used for ANOVA and the results are shown in Table 7 (22). The results of ANOVA show that educational background and grade are without significant effect on the safety belief and behavior of college students (Sig. > 0.05), similar to the survey findings for the college students in United States from Blair et al. (17). In contrast, gender, hometown, one-child policy, major and driver's license have significant influence on the safety beliefs and safety behaviors of college students (Sig. < 0.05).

**Table 7:** Analysis of variance of influencing factors.

	**Project**	**Number of cases**	**Average value**	**Std. Deviation**	**Std. Error**	**ANOVA**		
						***F***	**Sig**.	**Compare**
Gender	Male	2,388	155.45	22.991	0.470	4.151	0.042	Female>Male
	Female	2,090	156.92	25.378	0.555			
Education	Junior college	996	155.37	25.513	0.804	0.659	0.518	*P* > 0.05
background	Undergraduate	3,312	156.34	23.762	0.413			
	Postgraduate	170	156.61	23.140	1.791			
Grade	Freshman	1,322	155.68	24.298	0.668	2.389	0.067	*P* > 0.05
	Sophomore	1,773	156.72	24.426	0.580			
	Junior	1,103	156.58	23.750	0.715			
	Senior	280	152.82	22.923	1.370			
Hometown	Provincial capital	835	159.45	23.495	0.813	7.439	0.000	Provincial capital>County/prefecture>Non-provincial capital city>countryside
	Non provincial capital city	755	155.43	24.237	0.882			
	County/prefecture level city	1,563	156.06	24.681	0.624			
	Countryside	1,325	154.54	23.669	0.650			
Only child or not	Yes	1,889	157.4	24.622	0.567	8.934	0.003	Yes>No
	No	2,589	155.21	23.750	0.467			
Major	Science	2,683	156.83	24.755	0.672	5.199	0.039	Science>Liberal arts
	Liberal arts	1,795	155.15	23.472	0.554			
Community	MM-C2	1,586	157.76	23.886	0.599	4.879	0.000	MM-C2>MM-C1 >SS-C >A-C >PPE-C >LAS-C
	MM-C1	698	157.3	24.828	0.939			
	SS-C	527	156.87	25.223	1.099			
	A-C	321	154.13	24.002	1.34			
	PPE-C	877	154.01	22.14	0.751			
	LAS-C	469	153.4	25.833	1.192			
Driver's license	Have	1,587	158.43	24.235	0.608	17.737	0.000	Have> During the study> Don't have
	In the study	1,217	156.78	23.872	0.684			
	Do not have	1,674	153.49	24.010	0.587			

Regarding gender, it is found that female students show better safety belief and behavior than male students, which is consistent with the survey results among college students in United States from Crowe (23) and in Bengal from Hasan and Younos (21). This fact may result from the difference in the characteristic between female and male students. Universities should provide safety education in the ways that can attract the interest of male students, such as the mobile-phone application or short video production competition of safety education.

From the comparison of average value, the hometown differences of college students lead to the different in their understanding of safety knowledge and the ways of dealing with safety problems. Generally, the students from developed cities could receive a better level of safety education, not only resulting from the fact that their family are normally in a good financial status, but also more safety problems would be encountered in developed cities. These results regarding hometown are also consistent with those from Hasan and Younos (21).

Regarding on the one-child policy, the respondents from only-child families show better safety culture than those having sibling(s). Since most of the one-child families are in the city, the effect of one-child policy is kind of in accordance to the effect of hometown in the questionnaire. In addition, parents of one-child families would pay more attention to the safety education of their child.

Regarding major, it is found that the students who major in science and have more safety knowledge shows better safety culture than the students majoring in liberal arts. Gong (24) also believes that medical students who have more safety knowledge from their daily learning would show better safety beliefs and safety behaviors than other major students. Therefore, it is suggested that the university should strengthen the development and diversity of safety culture courses, so as to increase the safety knowledge of each student and effectively avoid safety problems.

**Figure 3:**
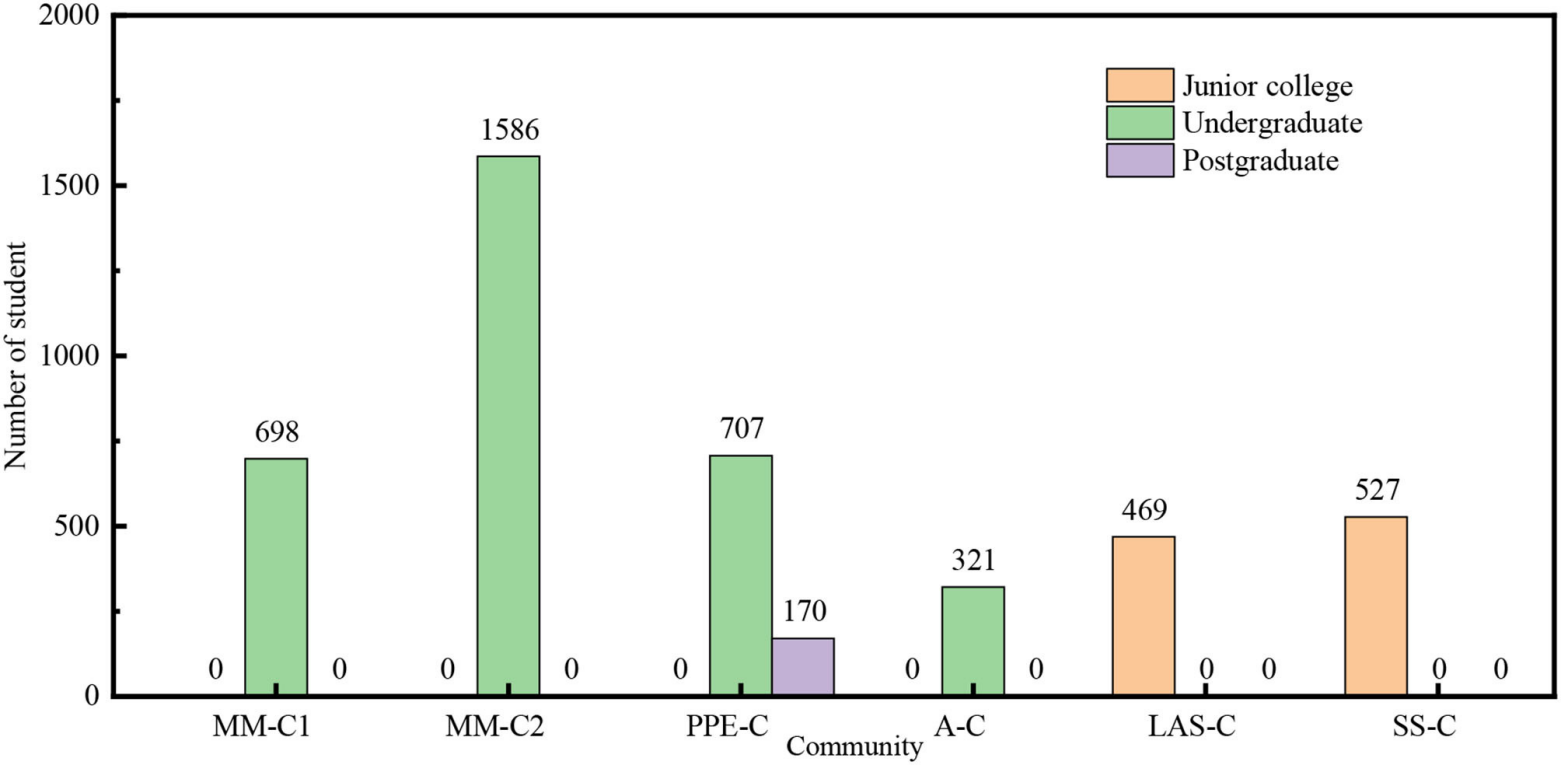
Educational background distribution in the community.

**Figure 4:**
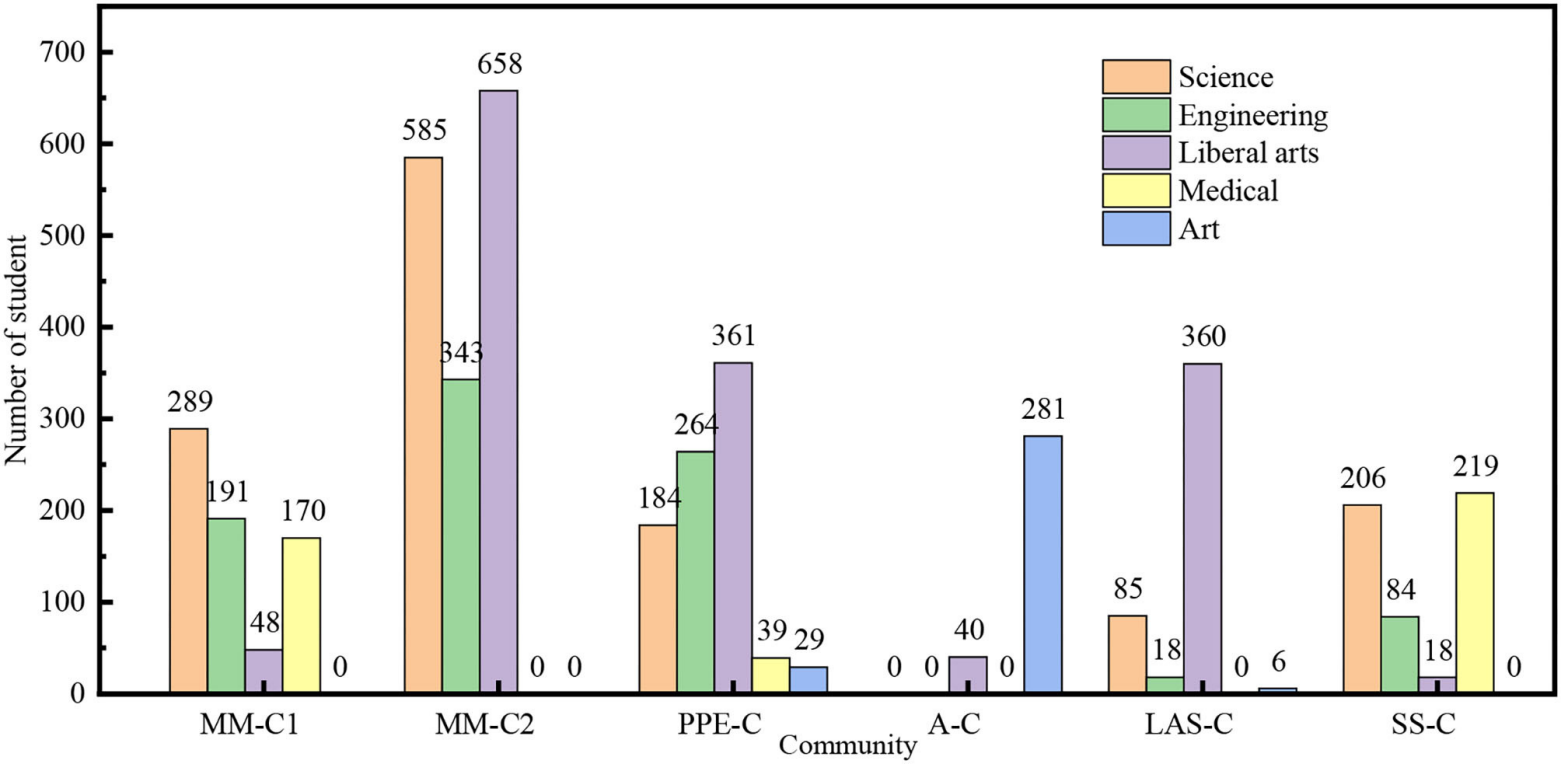
Major distribution in the community.

The advantage of community system is to break the boundary of major, and strengthen the communication among the students in different majors and cultural backgrounds. As shown in Table 7, the safety culture of the students from SS-C (Science specialty community of junior college) is much higher than that of the students from LAS-C(Liberal arts specialty community of junior college), since medical students in SS-C account for the majority as shown in Figures 3, 4. MM-C1 (Major-Mixed community 1), M-C2 (Major-Mixed community 2), and PPE-C (Community preparing for the postgraduate entrance test) are all mixed major communities. The students from MM-C1 and MM-C2 show better safety belief and safety behavior than those from PPE-C. The reason may be that the education of PPE-C particularly focuses on the postgraduate entrance test, neglecting safety education. Obviously, the community management system has a significant impact on the safety culture of college students.

Regarding driver's license, students who have obtained driver's license get much higher score than others. Before the driving test, people usually have a weak understanding of the traffic laws and regulations. During the study for driver's license, the safety belief and safety behavior of people would be greatly improved by learning the traffic laws and regulations. It indicates that the study of safety knowledge is very important to the cultivation of safety culture.

## Recommendations

Based on the analysis of the results, it is recommended that safety culture measurement should be conducted before freshman enrollment and repeated every year since then to improve the safety administration and education procedure for university students. A dynamically updated database for the questionnaire items should be also developed. Specific methods for improving safety culture should be adopted for specific students. In the development of intervention strategies to prevent safety problems of the college students, more attention should be paid to the safety culture cultivation of male students. Considering that the safety culture level of medical students is generally high, it is suggested that the general safety course should be strengthened and diversified in daily teaching. The community management system also should be optimized to give full play to its advantages in the safety culture cultivation of college students.

Even though this measurement deals with pretty large sample size and considers more factors which have never been reported in previous studies, such as Community, One-child policy and Driver's license, there are still some limitations of this study. The results of this measurement tool may show some deviation when it is used in more developed areas of China. After the abundance of One-child policy in China, this factor would show less and less influence on the safety culture of undergraduates.

## Conclusions

In this study, 4,531 students from a private university in Shaanxi province, China responded to the questionnaire anonymously online. By means of ANOVA, the influence of eight factors including education background, grade, hometown, one-child policy, major, community and driver's license, on their safety beliefs and safety behaviors was studied.

According to the average score of each item, the investigated private university students are lack of safety knowledge, but perform well in traffic safety behavior. Gender, hometown, one-child policy, major, community and driver's license have significant influence on the safety beliefs and safety behaviors of the respondents, but education background and grade do not. Female student has better safety beliefs and safety behaviors than male student. Since medical students have received the training of safety culture in their daily learning, their safety beliefs and safety behaviors are better than that of other major students. The safety beliefs and safety behaviors of only child families and students who have obtained driver's license are better than those of others. Under the community management system, each community has different major composition and cultural atmosphere, which is conducive to the formation of good safety beliefs and safety behaviors of students. The results highlighted that universities should formulate the corresponding intervention strategies to prevent safety problems of the college students according to the specific proportion of gender, major distribution and other actual situation.

To sum up, university safety administration and education is the key mean to improve risk avoidance ability of the college students and enhance their safety beliefs and safety behaviors. Universities should strengthen the regular safety knowledge popularization and various disaster emergency drills to improve the risk avoidance ability of college students.

## Data Availability Statement

The original contributions presented in the study are included in the article/Supplementary Material, further inquiries can be directed to the corresponding author/s.

## Ethics Statement

Approval was obtained from the Ethical Review Board of Xijing University. The procedures used in this study adhere to the tenets of the Declaration of Xijing University.

## Author Contributions

SG: investigation, writing—reviewing and editing, and methodology. FY: investigation and writing—reviewing and editing. FR: conceptualization. CC: data curation and writing—original draft preparation. All authors contributed to the article and approved the submitted version.

## Conflict of Interest

The authors declare that the research was conducted in the absence of any commercial or financial relationships that could be construed as a potential conflict of interest.

## Publisher's Note

All claims expressed in this article are solely those of the authors and do not necessarily represent those of their affiliated organizations, or those of the publisher, the editors and the reviewers. Any product that may be evaluated in this article, or claim that may be made by its manufacturer, is not guaranteed or endorsed by the publisher.

## Footnotes

^1^Chinacourt.org. *The Death Toll from the Fire at the Peoples' Friendship University of Russia Rose to 41* (2003). Available online at: https://www.chinacourt.org/article/detail/2003/12/id/95301.shtml (accessed December 10, 2003).

^2^CCTV.com. *The Alarm Rings Again: Issues Brought About by the “4.16” Shooting on American University Campuses* (2007). Available online at: http://news.cctv.com/world/20070418/102677.shtml (accessed April 4, 2007).

^3^Sohu.com. *One Dead and Five Injured in Laboratory Explosion at China University of Mining and Technology* (2015). Available online at: http://news.sohu.com/20150408/n410930329.shtml (accessed April 8, 2015).

## References

[1] Dong JY Ma CG Fu G Du C. Analysis and countermeasures for behavioral causes of university laboratories safety accidents. Exp Technol Manage. (2016) 33:258–61. 10.16791/j.cnki.sjg.2016.10.065

[2] Jing YJ Wu Y. Investigation and practice on current situation of university students' awareness of laboratory safety. Exp Technol Manage. (2019) 36:251–67. 10.16791/j.cnki.sjg.2019.05.059

[3] Feng LY Wang J Zhang Y Wang WK Liu Y. Study on influence mechanism and safety management of unsafe factors in universities based on social network. J Saf Sci Technol. (2020) 16:156–61. 10.11731/j.issn.1673-193x.2020.04.025

[4] Yang FQ Li W. Application of accident model and process in safety management of university laboratories. Res Explorat Lab. (2020) 39:285−32.19298799

[5] Walters AUC Lawrence W Jalsa NK. Chemical laboratory safety awareness, attitudes and practices of tertiary students. Saf Sci. (2017) 96:161–71. 10.1016/j.ssci.2017.03.017

[6] Wu TC Liu CW Lu MC. Safety climate in university and college laboratories: Impact of organizational and individual factors. J Saf Res. (2007) 38:91–102. 10.1016/j.jsr.2007.01.003 17303169

[7] Qin S. Research on influencing factors of social practice safety risk in colleges and universities. China Saf Sci J. (2021) 31:18–23. 10.16265/j.cnki.issn1003-3033.2021.01.003

[8] Li XR Xu SC Chen XB Sun Q. Analysis of the influence factors affecting campus security based on SEM model. Comput Appl Chem. (2018) 35:910–9. 10.16866/j.com.app.chem201811005

[9] Thamrin Y Pisaniello D Stewart S. Time trends and predictive factors for safety perceptions among incoming South Australian university students. J Saf Res. (2010) 41:59–63. 10.1016/j.jsr.2009.11.003 20226952

[10] Reesi HA Maniri AA Kai P Hinai MA Adawi SA Davey J. Risky driving behavior among university students and staff in the Sultanate of Oman. Accident Anal Prevent. (2013) 58:1–9. 10.1016/j.aap.2013.04.021 23689200

[11] Wang B Wu C. Safety culture development, research, and implementation in China: an overview. Prog Nuclear Energy. (2019) 110:289–300. 10.1016/j.pnucene.2018.10.00235281902

[12] Cronbach LJ. Coefficient alpha and the internal structure of tests. Psychometrika. (1951) 16:297–334. 10.1007/BF02310555

[13] Nunally JC Bernstein IH. Psychometric Theory. 2nd ed. New York, NY: McGraw-Hill Education, Inc. (1978).

[14] DeVellis RF. Scale Development: Theory and Applications. 3nd ed. London: Sage Publications (2011).

[15] Chou LP. Social Research Methods. 2nd ed. Chongqing: Chongqing University Press (2015).

[16] Kaiser HF Rice J. Little Jiffy, Mark IV. Educ Psychol Meas. (1974) 34:111–7. 10.1177/001316447403400115

[17] Blair EH Seo DC Torabi MR Kaldahl MA. Safety beliefs and safe behavior among Midwestern college students. J Saf Res. (2004) 35:131–40. 10.1016/j.jsr.2003.11.003 15178231

[18] Wei XL. The Impact of Stigma on Psychological Help-Seeking Willingness. Soochow University (2017).

[19] Guo T Zhu YS. A study on stigmatization of College Students' psychological help seeking. China J Multimedia Netw Teach. (2019) 3:122−3.

[20] Li ZY Wang Y Fang DH. Research and exploration of laboratory safety culture construction in university. Exp Technol Manage. (2021) 38:289–92. 10.16791/j.cnki.sjg.2021.02.06334904888

[21] Hasan MK Younos TB. Safety culture among Bangladeshi university students: a cross-sectional survey. Saf Sci. (2020) 131:104922. 10.1016/j.ssci.2020.104922

[22] Lu XG. Practice Tutorial for Social Survey Research Based on SPSS 20. Beijing: Posts and Telecommunications Press (2016).

[23] Crowe JW. Safety beliefs and safe practices among college students. J Saf Res. (1995) 26:187–95. 10.1016/0022-4375(95)00010-N

[24] Gong YH. Safety culture among Chinese undergraduates: a survey at a university. Saf Sci. (2019) 111:17–21. 10.1016/j.ssci.2018.09.010

