# Supplementary material for: Safety Culture Measurement Among Chinese Undergraduates at a Private University: Development and Validation
Source: Front Public Health. 2022 Mar 28;10:825106. doi: 10.3389/fpubh.2022.825106 (PMC8995428; doi:10.3389/fpubh.2022.825106)
Supplement: Supplementary file 1 [file Data_Sheet_1.pdf]

Appendix A

Table A. 1 Commonality of questionnaire validity test

Item	Initial	Extract	Item	Initial	Extract
QA1	1.000	0.562	QX1	1.000	0.485
QA2	1.000	0.643	QX2	1.000	0.541
QA3	1.000	0.55	QX3	1.000	0.544
QA4	1.000	0.697	QX4	1.000	0.547
QA5	1.000	0.778	QX5	1.000	0.537
QA6	1.000	0.78	QX6	1.000	0.508
QA7	1.000	0.7	QX7	1.000	0.601
QA8	1.000	0.561	QX8	1.000	0.569
QD1	1.000	0.627	QX9	1.000	0.603
QD2	1.000	0.635	QX10	1.000	0.439
QD3	1.000	0.691	QX11	1.000	0.548
QD4	1.000	0.519	QX12	1.000	0.625
QD5	1.000	0.565	QX13	1.000	0.538
QD6	1.000	0.635	QX14	1.000	0.53
QD7	1.000	0.567	QX15	1.000	0.517
QD8	1.000	0.497			
QJ1	1.000	0.654			
QJ2	1.000	0.703			
QJ3	1.000	0.697			
QJ4	1.000	0.521			
QJ5	1.000	0.638			
QJ6	1.000	0.536			
QJ7	1.000	0.631			
QJ8	1.000	0.545			

Extraction Method: Principal Component Analysis.

Table A. 2 Questionnaire validity test total variance explained

	Initial Eigenvalues			Extraction Sums of Squared Loadings			Rotation Sums of Squared Loadings		
Component	Total	% Of Variance	Cumulative %	Total	% Of Variance	Cumulative %	Total	% Of Variance	Cumulative %
1	16.156	41.425	41.425	16.156	41.425	41.425	8.032	20.595	20.595
2	3.014	7.727	49.152	3.014	7.727	49.152	4.953	12.7	33.295
3	1.613	4.136	53.288	1.613	4.136	53.288	3.616	9.273	42.568

4	1.229	3.15	56.438	1.229	3.15	56.438	3.354	8.601	51.168
5	1.052	2.699	59.137	1.052	2.699	59.137	3.108	7.968	59.137
6	0.948	2.431	61.568						
7	0.798	2.045	63.613						
8	0.75	1.923	65.536						
9	0.718	1.841	67.376						
10	0.674	1.727	69.104						
11	0.646	1.657	70.761						
12	0.611	1.565	72.327						
13	0.591	1.517	73.843						
14	0.572	1.467	75.311						
15	0.537	1.376	76.687						
16	0.51	1.309	77.996						
17	0.499	1.278	79.274						
18	0.484	1.241	80.515						
19	0.473	1.213	81.727						
20	0.462	1.183	82.911						
21	0.453	1.162	84.073						
22	0.445	1.14	85.213						
23	0.441	1.132	86.345						
24	0.421	1.08	87.425						
25	0.413	1.058	88.483						
26	0.401	1.029	89.512						
27	0.392	1.005	90.517						
28	0.386	0.989	91.506						
29	0.369	0.947	92.453						
30	0.363	0.931	93.384						
31	0.355	0.911	94.295						
32	0.346	0.888	95.183						
33	0.337	0.863	96.046						
34	0.327	0.838	96.884						
35	0.304	0.779	97.662						
36	0.293	0.75	98.413						
37	0.247	0.632	99.045						
38	0.237	0.607	99.652						
39	0.136	0.348	100						

Extraction Method: Principal Component Analysis.
